# Editorial: Effects of Plant-Microbiome Interactions on Phyto- and Bio-Remediation Capacity

**DOI:** 10.3389/fpls.2019.00533

**Published:** 2019-04-26

**Authors:** Angela Cicatelli, Nuria Ferrol, Piotr Rozpadek, Stefano Castiglione

**Affiliations:** ^1^Department of Chemistry and Biology “A. Zambelli”, University of Salerno, Fisciano, Italy; ^2^Department of Soil Microbiology and Symbiotic Systems, Estación Experimental del Zaidin, CSIC, Granada, Spain; ^3^Plant-Microorganism Interactions, Małopolska Centre of Biotechnology, Jagiellonian University, Kraków, Poland

**Keywords:** bio-remediation, interaction, PGPR, endophytes, mycorrhiza, NGS, constructed wetlands

## Introduction

Bio-remediation is a process that looks at plants and microorganisms (bacteria and fungi) as natural systems that are able to degrade, transform, and even accumulate large quantities of contaminants that are found, naturally or have been introduced into the environment. Emerging evidence indicates that this is possible thanks to strong plant-microorganism interactions that take place preferentially at soil level (Masciandaro et al., 2013).

In the past, plants and microorganisms have been employed separately for bio-remediation. However, in recent years, several studies have demonstrated that they can act synergistically to improve the remediation process of different matrices, such as water, soil, and air (Khan et al., 2018). The rhizosphere is the thin layer of soil (1–2 mm) adherent to the rhizoplane where the majority of soil microbiota resides (Walker et al., 2003). Plants release large amounts of photosynthesis byproducts (up to 30%) into the soil that nourish rhizosphere microorganisms (Canarini et al., 2019). Microorganisms in turn are able to improve cation exchange capacity (CEC) altering soil water pH, and release siderophores and phosphatases, which improve plant nutrition, and even secrete enzymes that are able to reduce plant stress, such as ACC deaminase (Pilon-Smits, 2005). Some fungi have established a broad, strong, and fundamental relationship with plants for more than 400 million years, the so-called endo- and ecto-mycorrhizal symbioses (Remy et al., 1994). Endomycorrhiza have been used to improve phosphorus removal using trees (e.g., willow and poplar) as vegetation filters (Fillion et al., 2011). While other fungi and bacterial species live inside plant tissues and are defined for this characteristic as “endophytes” (Zhang et al., 2019).

Plants and microorganisms have been recently used to ameliorate different phytoremediation processes, such as phyto-extraction (Sessitsch et al., 2013; Vigliotta et al., 2016), phyto-degradation (Feng et al., 2017), and phyto-stimulation (Cicatelli et al., 2017). All these different strategies are fundamental for reclamation of polluted water by means of the constructed wetlands (CWs), a very promising application of bio-remediation developed in the last decade (Wu et al., 2015). In fact, in CWs plants and microorganisms fruitfully interact in the bio-remediation of urban, livestock and industrial wastes. The aim of this research topic was to highlight this specific field of Plant Sciences which, even though very promising, is still in its infancy. The research articles published here address many aspects of bio-remediation, but they all provide evidence that isolated bacteria and fungi, growing in different soils, contaminated or not, can help plants to survive in harsh environments that are often polluted by organic compounds or by inorganic elements. In some of the articles published in this eBook, Next Generation Sequencing (NGS) techniques (Derocles et al., 2018) have been applied to study the rhizosphere microbiome. These molecular tools, introduced within the last decade, have not yet fully reached their potential; however, already they allow scientists to discover new and unknown taxa of microorganisms.

The articles published in this eBook focus on plant-bacteria and/or plant-fungi interactions, and the effects that soil pollutants have on the plant microbiota.

Root bacterial communities of several plant species were investigated with the goal of understanding how certain pollutants, present in the soil or water, affect them in terms of biodiversity and composition. Five of the articles of the eBook illustrate how many bacterial strains, belonging to different species or taxa (e.g.*, Bacillus, Serratia*, etc.), and from which plant species, growing on different soil conditions, have been isolated from the rhizospere. Several of these bacteria strains showed resistance to inorganic pollutants of natural (Caneschi et al. - Brazilian Ironstone) and/or anthropogenic origin (Mesa-Marín et al. - heavy metals, Zappellini et al.- red gypsum landfill), or to organic compounds (Thijs et al. - TNT, Cao et al. - atrazine). The goal of the research reported in these articles was that to identify the ones showing plant growth promoting characters (PGP-Kloepper and Schroth, 1978) in the presence of soil pollutants (Segura et al., 2009). All of the identified bacterial strains were biochemically characterized for their PGP capabilities, such as to solubilise inorganic phosphate, or to produce siderophores, organic acids, IAA, and/or other compounds. The isolates were also molecularly profiled using hyper-variable regions of the 16S rDNA in order to relate them phylogenetically to known bacteria for which these sequences have been already deposited in public databases. Some bacterial strains, either individually or in consortium, exhibited high bio-remediating potential. In particular, they were shown to: (i) reduce plant stress in harsh environments; (ii) increase the uptake, translocation, accumulation, and/or modification of the contaminants to the aerial parts.

Some of the aforementioned articles demonstrated that the addition of microorganisms to the polluted soil had a significant effect on plant biomass production, which is ultimately the most indicative parameter to evaluate the efficacy of the bio-remediation treatment.

Three of the articles published here deal with the effect of mycorrhization on the ability of forest trees (Nadeau et al. - White spruce, Gil-Martínez et al. - Holm Oak) to counteract metal toxicity. Plants were exposed to pollutants of different origin including: trace elements (TE), heavy metals, as in the case of waste rocks (WRs), or fine tailings (FTs) commonly present in abandoned mine sites. Mycorrhization, in particular ecto-mycorrhization, occurs in woody pioneer species such as those mentioned above. Holm oak, however, cannot be considered a forest pioneer but rather a late successional species. Ecto-mycorrhiza improve phosphorus and nitrogen uptake by trees and enhance resistance to diverse stress factors, such as drought, salinity, heavy metals, and pathogens. As in the case of PGPRs, fungal communities are also strongly modified by soil contaminants; in fact, environmental constraints lead to the selection of specific and environmental well-adapted taxa.

Two of the 11 articles deal with endophytic bacteria or fungi. Their presence in herbaceous plants grown in soil containing organic pollutants (Lumactud and Fulthorpe - petroleum hydrocarbons), or metalloids (Lindblom et al. - *Alternaria tenuissima* and selenium) was studied in detail. In the case of endophytic bacteria, it was confirmed once again, as for rhizosphere bacteria, their PGP character and their ubiquity, since they were present in different plant species growing on the same contaminated site. Even though fungi were the first endophyte to be discovered at the beginning of the nineteenth century (De Bary, 1866), little is still known about plant-fungus interactions. Although the article published here on this topic refers to an unusual case (a fungal endophyte of a selenium hyperaccumulator), it confirmed that fungal colonization of plant tissues, organs and reproductive structures (seeds) favors the host survival due to the production of compounds that protect them from high contents of metals or metalloids present in the soil. The endophytes can also protect plants from herbivores, and increase nutrient absorption and biomass.

An interesting application of bio-remediation is that of the Constructed Wetlands. Similar to other bio-remediation strategies, which include bio-degradation, bio-stimulation and bio-augmentation, both rhizosphere and/or endophytic microorganisms can be utilized in establishing CWs. In fact, they have been widely studied, and, in many CWs, the synergic activities of plants and microorganisms (Syranidou et al. - CWs, emerging pollutants and bio-augmentation) have been clearly demonstrated. Therefore, CWs can be considered a realistic alternative to the more traditional techniques employed to reclaim waters polluted by various compounds introduced into the environment by urban, agricultural, and industrial activities.

## Author Contributions

SC is responsible for organizing the materials and writing the editorial. AC, NF, and PR are responsible for its reading and revising.

### Conflict of Interest Statement

The authors declare that the research was conducted in the absence of any commercial or financial relationships that could be construed as a potential conflict of interest.
